# Molecular diagnosis of cytomegalovirus, Epstein-Barr virus and Herpes virus 6 among blood donors in Ouagadougou, Burkina Faso

**DOI:** 10.1186/1471-2334-14-S2-P99

**Published:** 2014-05-23

**Authors:** Lassina Traore, Issoufou Tao, Cyrille Bisseye, Florencia Djigma, Djénéba Ouermi, Theodora Zohoncon, Tegwindé Rebecca Compaoré, Birama Diarra, Maleki Asshi, Jacques Simpore

**Affiliations:** 1 Labiogene, Ouagadougou, Burkina Faso

## Introduction

This study focuses on three herpes viruses, including EBV, CMV and HHV -6. Our study aims to determine the prevalence of these viruses in blood donors in Ouagadougou.

## Material and methods

The study included 198 blood donors. We extracted DNA, using DNA extraction kit -Sorb -B. For amplification, we used PCR Real -96 SaCycler of V.7.3 "Sacace Biotechnology" and kit As CMV/EBV/HHV-6 TM Real - Time apparatus. The results were analyzed using the standard software SPSS -17 for Windows and EpiInfo-6 -6.

## Results

Of the 198 samples tested, 18 (9.09%) were positive to at least one of the three viruses, 10 (5.10%) were positive for EBV, 10 (5.10%) positive for CMV and 12 (6.10%) positive for HHV -6. According to age, we found that only those who had a less than or equal to 30 years old were infected. Infection with EBV, CMV and HHV-6 in women accounted for 8.57%, 8.57% and 11.43% respectively. Against by men, infection rates were low were 4.29%, 3.68% and 4.90 % for EBV, CMV and HHV -6. Based on HIV status we found that HIV-positive were more infected than HIV-negative, EBV (12.5 % versus 4.74), CMV (12.5 % versus 4.74) , HHV-6 (12.5% versus 5.79) . Seven (7) samples were co- infected EBV/CMV/HHV-6, or 38.89 % of positive samples.

## Conclusion

The prevalence that we recorded is low compared to those reported by previous studies in the sub-region among blood donors. This difference can be explained by the fact that previous studies have used serological techniques.

